# High throughput chemical library screening identifies a novel p110-δ inhibitor that potentiates the anti-myeloma effect of bortezomib

**DOI:** 10.18632/oncotarget.9568

**Published:** 2016-05-24

**Authors:** Ehsan Malek, James J. Driscoll

**Affiliations:** ^1^ Case Western Reserve University School of Medicine, Cleveland, OH, USA; ^2^ Division of Hematology and Oncology, University of Cincinnati College of Medicine, Cincinnati, OH, USA; ^3^ The Vontz Center for Molecular Studies, University of Cincinnati College of Medicine, Cincinnati, OH, USA; ^4^ University of Cincinnati Cancer Institute, Cincinnati, OH, USA

**Keywords:** phosphatidylinositol 3-kinase, p110-delta, multiple myeloma, proteasome, bortezomib resistance

## Abstract

Multiple myeloma (MM) remains an incurable plasma cell malignancy and drug resistance persists as the major cause of treatment failure leading to fatal outcomes. The phosphatidyl-inositol-3-kinase (PI3K) pathway is constitutively hyperactivated in MM to promote disease progression and drug resistance. While inhibiting PI3K induces apoptosis in MM and is predicted to increase tumor susceptibility to anticancer therapy, early-generation pan-PI3K inhibitors display poor clinical efficacy as well as intolerable side effects. Here, we found that PI3K activity is significantly upregulated in MM cell lines and patient tumor cells resistant to bortezomib and that the majority of PI3K activity in MM cells is dependent upon the p110-δ isoform. Genetic or pharmacologic inhibition of p110-δ substantially reduced myeloma viability and enhanced cellular sensitivity to bortezomib. Chemical library screens then identified a novel compound, DT97, that potently inhibited p110-δ kinase activity and induced apoptosis in MM cells. DT97 was evaluated in the NCI-60 panel of human cancer cell types and anticancer activity was greatest against MM, leukemia and lymphoma cells. Co-treatment with DT97 and bortezomib synergistically induced apoptosis in MM patient cells and overcame bortezomib-resistance. Although bone marrow stromal cells (BMSCs) promote MM growth, the pro-survival effects of BMSCs were significantly reduced by DT97 treatment. Co-treatment with bortezomib and DT97 reduced the growth of myeloma xenotransplants in murine models and prolonged host survival. Taken together, the results provide the basis for further clinical evaluation of p110-δ inhibitors, as monotherapy or in synergistic combinations, for the benefit of MM patients.

## INTRODUCTION

Despite recent advances in understanding myelomagenesis and the discovery of proteasome inhibitors (PIs) and immunomodulatory agents, the majority of multiple myeloma (MM) patients continue to have a relapse and MM remains nearly uniformly fatal [1–3]. Drug resistance is a common cause of poor patient outcome, however, the precise mechanisms that underlie drug resistance remain poorly defined and effective strategies to prevent or revert resistance are lacking [4–8]. The phosphatidyl-inositol-3-kinase (PI3K) pathway is hyperactivated in MM cells to create a state of kinase dependency, and consequently, exquisite sensitivity to certain PI3K inhibitors. Controlling key growth-promoting pathways with small molecule drugs is an emerging strategy that can improve treatment of MM patients.

Class I PI3Ks consist of regulatory subunit (p85 or p101) bound to a catalytic subunit of the class 1A (p110-α, −β, and −δ) or class 1B (p110-γ) isoforms [9]. The genes *PIK3CA, PIK3CB, PIK3CD* and *PIK3CG* express the PI3K/p110-δ, β, γ and δ isoforms. Expression of p110-γ is largely restricted to leukocytes, whereas the expression of p110-α and p110-β appears ubiquitous. *PIK3CA, PIK3CB, PIK3CD* or *PIK3CG* gene mutations in MM cells have not been reported [10–12]. PI3K inhibitors have shown promise in mouse models of cancer and led to the development of multiple agents currently being evaluated in clinical trials. The PI3K isoforms appear to fulfill distinct roles during physiologic and pathologic conditions, suggesting that isoform-specific inhibitors may more target tumor growth [13, 14]. Moreover, pan-PI3K inhibitors have not been successful in clinical studies and have yielded numerous adverse effects in patients. Therefore, inhibitors that are selective for a single PI3K isoform may offer more refined activity with reduced adverse effects. p110-δ has a crucial role in a plethora of leukocyte and B cell functions, including proliferation, antibody secretion, survival and migration [15–18]. Genetic or pharmacologic inactivation of p110-δ demonstrates its critical importance in B-cell signaling and survival [19–23].

We sought to identify small molecules that inhibited p110-δ activity and potentiated the anti-myeloma effect of bortezomib. Our studies were fueled by the remarkable success of the FDA-approved p110-δ inhibitor idelalisib (Zydelig^®^) that exhibits significant activity for the treatment of chronic lymphocytic leukemia (CLL), relapsed of follicular non-Hodgkin's lymphoma (NHL) or small lymphocytic lymphoma (SLL) [19]. However, idelalisib is not effective in treating MM and can generate numerous severe, adverse effects [21]. Development of p110-δ inhibitors that overcome the drawbacks associated with current p110-δ-targeting drugs and that are effective in MM patients represents an urgent and unmet need.

## RESULTS

### PI3K activity is increased in PCs from MM patients relative to those from healthy individuals or MGUS patients

**Figure 1:**
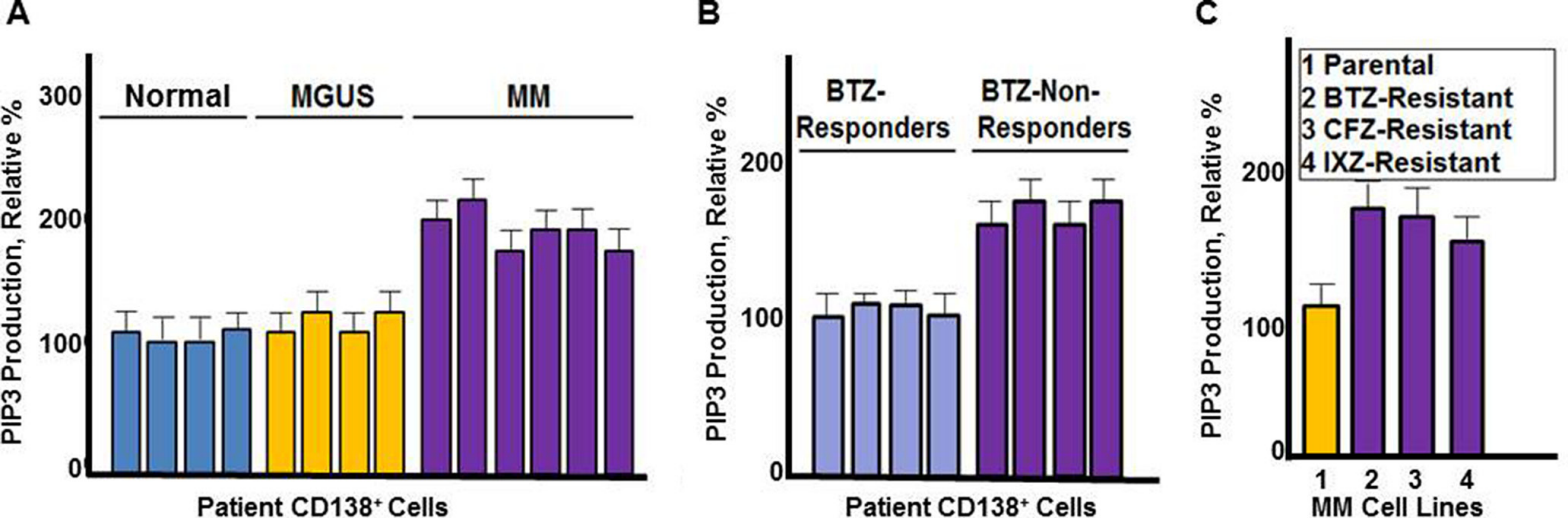
PI3K catalytic activity in MM cells (**A**) Comparison of healthy (normal), MGUS and MM CD138^+^ cells. PIP3 production was measured using CD138^+^cells from healthy individuals, MGUS or MM patients. Cells were incubated with substrate and the amount of PIP3 generated quantitated by ELISA according to the manufacturer's instructions. (**B**) PIP3 production from CD138^+^ cells of MM patients that were either clinical responders or non-responders to bortezomib-based therapy. (**C**) PIP3 production from bortezomib sensitive and resistant MM cells. Each assay contained approximately 10,000 cells. All assays were performed in triplicate, values shown represent the mean and error bars represent the SD.

The contribution of PI3K kinase activity in MM remains poorly understood. To investigate the role of PI3K, we directly measured PI3K kinase activity in CD138^+^ cells that had been isolated from healthy individuals, monoclonal gammopathy of unknown significance (MGUS) or MM patients (Figure 1A). MGUS is a pre-malignant condition that nearly uniformly precedes the development of MM. PI3K kinase activity was directly measured by quantitating production of phosphatidylinositol [3, 4, 5]-trisphosphate (PIP3) using a colorimetric ELISA assay. PI3K activity was greater in PCs from MM patients compared to PCs from MGUS or healthy individuals (Figure 1A).

### PI3K activity is increased in bortezomib-resistant MM cells

We then compared PI3K activity in CD138^+^ cells isolated from MM patients that did or did not respond to bortezomib treatment (Figure 1B). PI3K kinase activity was greater in CD138^+^ cells from bortezomib non-responders compared to bortezomib responders. RPMI8226 cells resistant to PIs were generated as described [38] and results indicated that PI3K activity was also greater in cells the cells resistant to PIs -resistant cells compared to those that were drug-naïve (Figure 1C).

### Genetic inactivation of p110-δ reduces MM growth and sensitizes cells to bortezomib

**Figure 2:**
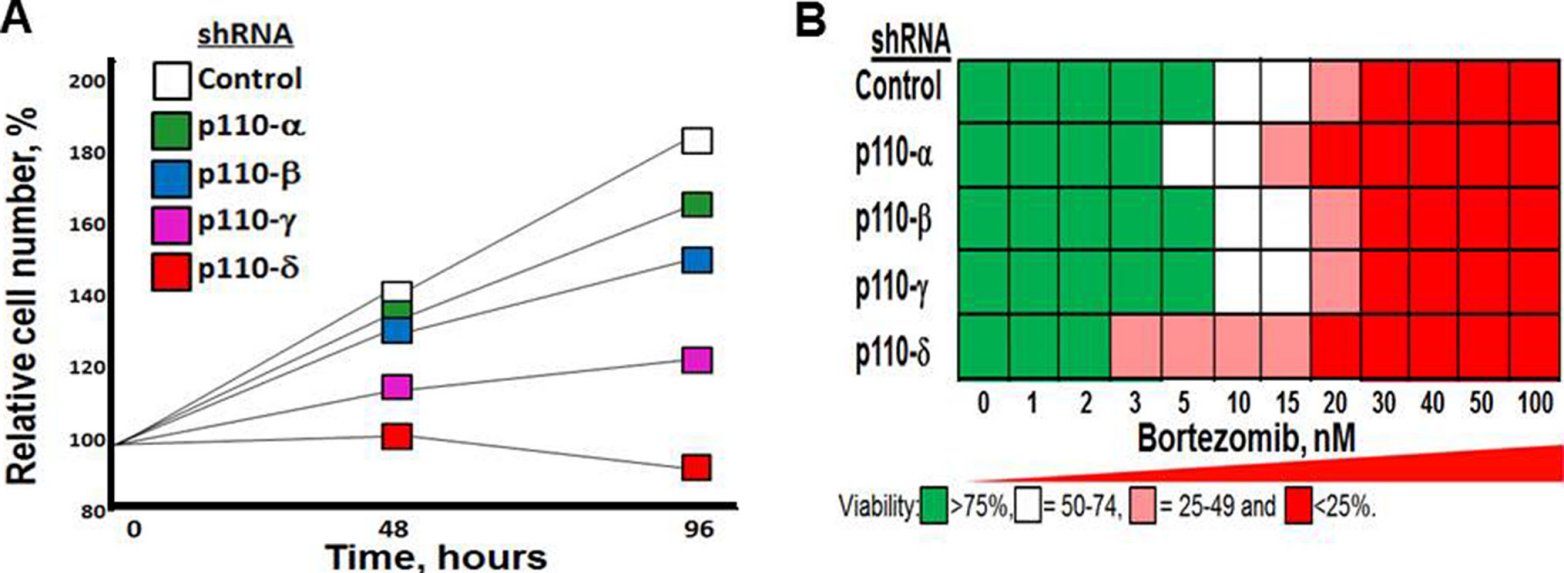
Effect of p110-δ knockdown on MM cell growth and bortezomib sensitivity (**A**) Effect of p110-isoform specific shRNA on MM cell growth. RPMI8226 cells were transfected as indicated, selected in puromycn and cell number counted at indicated times. (**B**) Effect of p110-isoform specific shRNA on MM cell sensitivtiy to bortezomib. Cells were transfected as indicated, treated with bortezomib and cell viability determined using the XTT assay.

We determined the effect of shRNA knockdown of individual PI3K isoforms on MM growth. RPMI8226 cells were transfected using shRNA to specifically inactivate the individual p110 isoforms. Knockdown of p110–δ most significantly reduced the growth rate of RPMI8226 MM cells (Figure 2A). The role of p110-δ has been studied *in vivo* in a p110-δ null mouse and in a p110-δ kinase-dead mouse (p110-δ^D910A/D910A^) [23]. Phenotypic analyses, *in vitro* and *ex vivo* studies determined that p110-δ is the predominant PI3K isoform that regulates the B cell phenotype. Moreover, using cells derived from p110-δ kinase-dead mice, it was shown that 90% of the PI3K kinase activity was dependent on p110-δ. Importantly, knockdown of *PIK3CD* also preferentially sensitized MM cells to proteasome inhibition (Figure 2B).

### High-throughput screening identifies a novel p110-δ inhibitor

**Figure 3:**
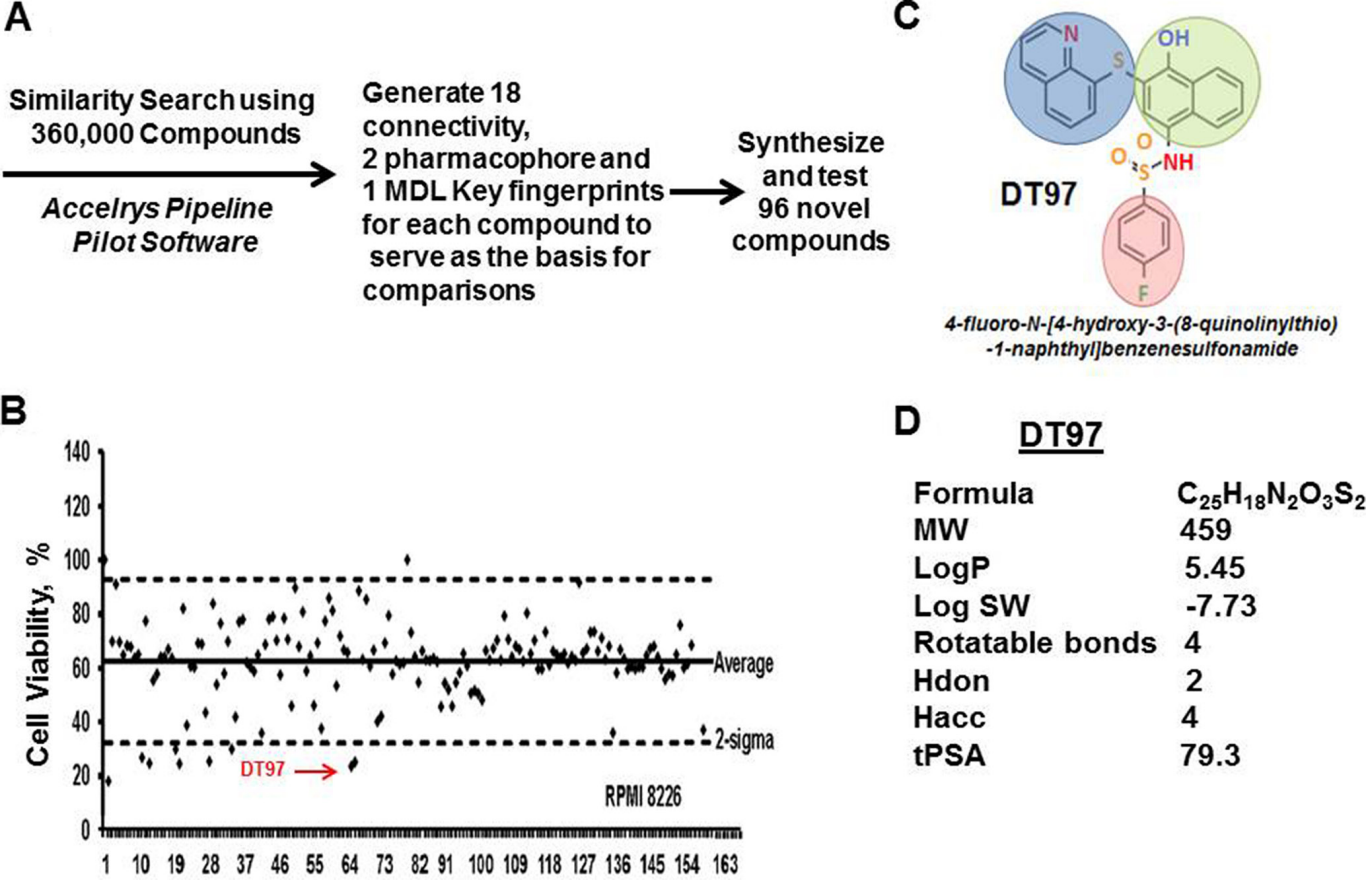
High-throughput screening of chemical compounds to identify novel p110-δ inhibitors (**A**) Target structures were searched using 360,000 compounds in the drug discovery library and Accelrys Pipeline Pilot Software. Similarity searches were performed through comparison of molecular fingerprints. The Accelrys Pipeline Pilot (version 8.0.1.600) was used to generate 18 connectivity, 2 pharmacophore and 1 MDLKeys fingerprints for each compound to serve as the basis for comparisons. The two pharmacophore fingerprints were averaged due to strong redundancy, leaving a total of 21 fingerprint types for the analysis. These are circular fingerprints based on substructure environments of a specific length (2, 4, or 6 atoms) centered about each atom of the molecule. Each atom of the molecule generates such an integer string, with each integer representing an atom within x atoms of the initial point. The composite of these strings is a matrix of character strings representing the entire molecule, i.e., the aggregate of the parts is the whole). Different fingerprints have slightly different strengths and weaknesses, so the UC DDC executes each compound search as 21 parallel searches that are combined as shown in the graphic below. (**B**) Viability of MM cells after treatment with either the 96 compounds identified in the virtual screen or with known commercially-available PI3K inhibitors. (**C**) Chemical structure of DT97. (**D**) DT97 physico-chemical properties.

High-throughput screening (HTS) of chemical libraries represents a systematic and inexpensive method to rapidly identify novel compounds that specifically inhibit protein targets that promote or maintain tumor growth. Based upon the comparison of chemical structures for the pan-PI3K inhibitor GSK2126458 and the p110-δ-specific inhibitor CAL-101, we performed virtual screening to identify novel chemical compounds that potentially inhibited p110-δ kinase activity (Figure 3A). Virtual screening of 360,000 compounds in the University of Cincinnati Drug Discovery Library was performed using the Accelrys Pipeline Pilot Software. Similarity searches compared the molecular fingerprints of chemical compounds to those of the pan-PI3K and p110-δ inhibitors. The top 96 compounds identified in the screen were then obtained and tested for ability to reduce the viability of myeloma cells in a cell-based *in vitro* assay using RPMI8226 cells (Figure 3B). The anti-myeloma effect of the 96 compounds was simultaneously compared to a panel of 68 known PI3K/AKT/mTOR inhibitors. We identified the most potent compounds that exhibited anti-myeloma activity and that also reduced PIP3 production. In this manner, we identified several promising hits with diverse scaffolds that ultimately led to the identification of our lead compound, DT97 (Figure 3C). DT97, (N-[4-hydroxy-3-(8-quinolinylthio)-1-naphthyl]benzenesulfonamide), was the most potent compound that reduced myeloma viability, reduced PIP3 production and that also possessed favorable physico-chemical properties (Figure 3D).

### DT97 inhibits p110-δ kinase activity

**Figure 4:**
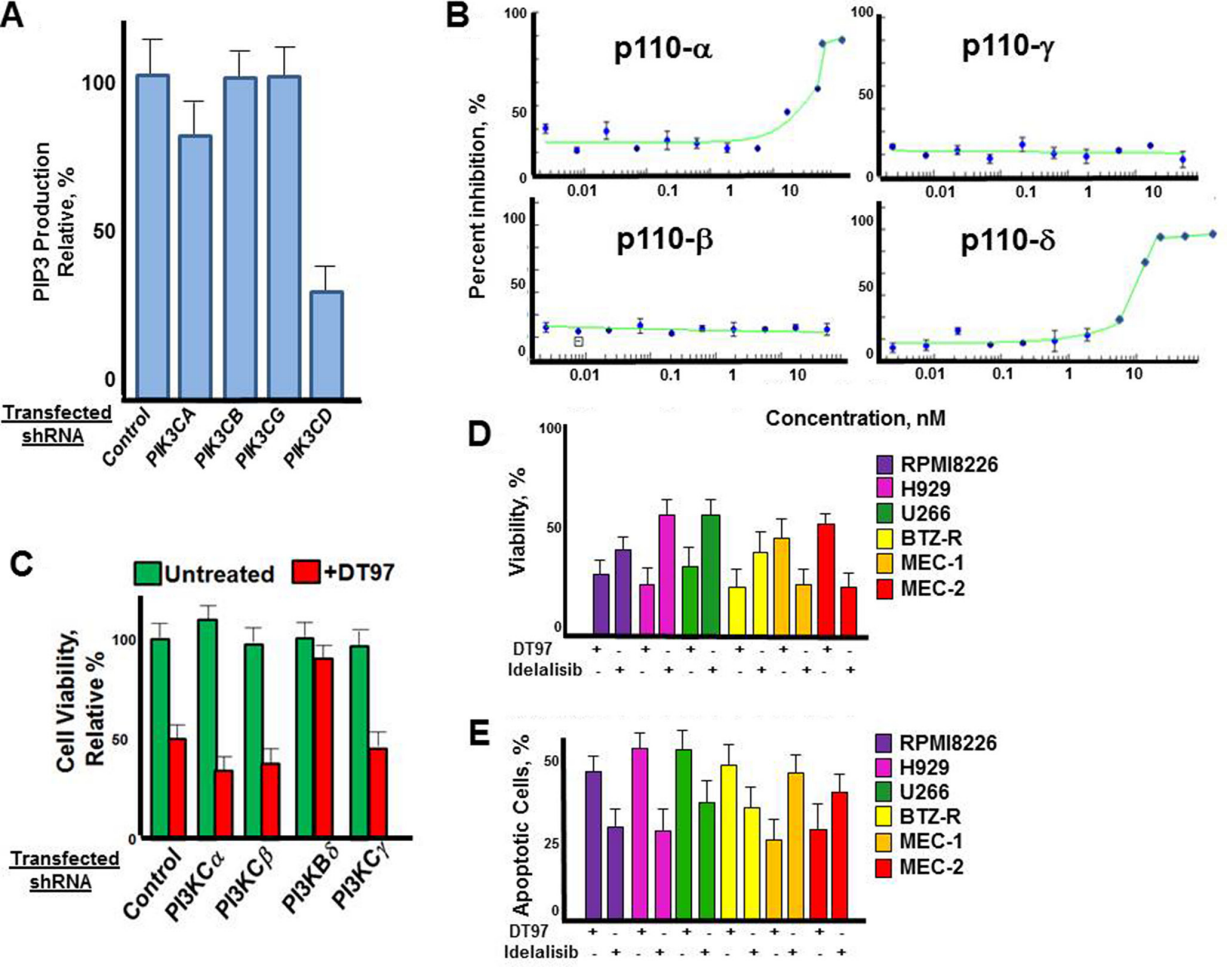
Effect of DT97 on p110-δ kinase activity (**A**) Effect of individual p110 isoform knockdown on PIP3 production. RPMI8226 cells were transfected with shRNA to scrambled control or isoforms *PIK3CA, PIK3CB, PIK3CG* or *PIK3CD*. PIP3 production was then determined from the transfected cells after puromycin selection. Data points represent the average value from triplicate measurements. (**B**) Dose-dependent effect of DT97 on purified p110-δ. Assays were performed in triplicate, values represent the mean and error bars represent the SD. (**C**) Effect of DT97 treatment on RPMI8226 cells after transfection with control shRNA or shRNA specific to individual PIK3C isoforms. Cells were transfected, selected in puromycin and cell viabiltiy determined using the XTT assay. Values represent the average of triplicate measurements. (**D**) Effect of DT97 and idelalisib on MM and CLL cell viability. The effect of each agent at 1 uM was determined using the XTT assay after treatment for 72 h. Values represent the average of triplicate measurements. (**E**) Effect of DT97 and idelalisib on apoptosis in MM and CLL cells. The effect of each agent at 1 uM was determined by counting the number of apoptotic cells after 24 h. Values represent the average of triplicate measurements.

We next determined the effect of individual p110 isoforms on PI3K kinase activity in RPMI8226 cells. Cells were transfected with isoform-specific shRNA to individually knockdown p110 isoforms, cultured in the presence of puromycin and PIP3 production then measured in the transfected cells (Figure 4A). Results indicated that the knockdown of p110-δ dramatically reduced PIP3 production in RPMI8226 cells, while knockdown of p110-α reduced PIP3 production by ~20%. Knockdown of p110-β and p110–γ had minimal effect on PIP3 production. The results were consistent with prior studies to indicated that the majority of PI3K activity in plasma cells (PCs) is dependent upon p110-δ [22, 23]. The dose-dependent effect of DT97 on PIP3 production by individual p110-isoforms was then determined (Figure 4B). The results indicated that DT97 inhibited the production of PIP3 most potently against the p110-δ isoform and had weaker activity against p110-α and no effect on p110-β or p110-γ. By comparison, idelalisib (formerly CAL-101) predominantly inhibits the p110-δ isoform but also reduces p110-γ activity (Table 1) [32]. DT97 did not significantly inhibit a number of other kinases that have been targeted to treat human cancers (Table 1).

**Table 1:** Inhibitory profile of DT97 and idelalisib

Kinase	DT97	Idelalisib
Class I PI3Ks		
p110-α	++	+
p110-β	none	+
p110-γ	none	++
p110-δ	+++	+++
**Class II PI3Ks**		
PKC2-α	none	none
PKC2β	+	+
PKCγ	none	none
		
**Class III PI3Ks**		
VPS34	none	+
		
**Class IV PI3Ks**		
DNA-PK	+	+
mTOR	+	+
		
**Other Clinically-relevant Kinases**		
FGFR	none	ND
PDGFR	none	none
ERBB2	+	none
ERBB4	+	none
MAPK	+	ND
AMPK	+	ND

Values for the effect of DT97 on kinases were obtained from 10-point concentration assays. Values for idelalisib were obtained from ref. 32. ND indicates that values were not determined. None indicates that significant activity was not detected. Plus signs indicate the activity relative to that of either DT97 or idelalisib.

To more rigorously define the on-target effect of DT97 on MM cytotoxicity, we then demonstrated that *PIK3CD* knockdown significantly reduced the effect of DT97 on the viability of RPMI8226 cells (Figure 4C). Treatment with DT97 had little effect on RPMI8226 cells after shRNA knockdown of *PIK3CD*. The *in vitro* growth inhibitory effect of DT97 on RPMI8226 cells was then determined and compared to that of idelalisib (Figure 4D). DT97 reduced the viability of parental and drug-resistant MM cells more potently than idelalisib. The p110-δ isoform is critical for transformation in CLL and activates the serine (Ser)–threonine (Thr) kinases AKT and mammalian target of rapamycin (mTOR). However, CXCR4, CD40 and CD49d, also play important roles in this process. Idelalisib is a potent, p110-δ inhibitor that is FDA-approved for the treatment of CLL. While idelalisib more effectively reduced the viability of CLL cells, DT97 more potently reduced the viability of MM cells. DT97 treatment generated more apoptotic MM cells than CLL cells while idelalisib had a more potent effect on CLL cells that DT97 (Figure 4E).

### DT97 overcomes bortezomib-resistance and inhibits chemotaxis

**Figure 5:**
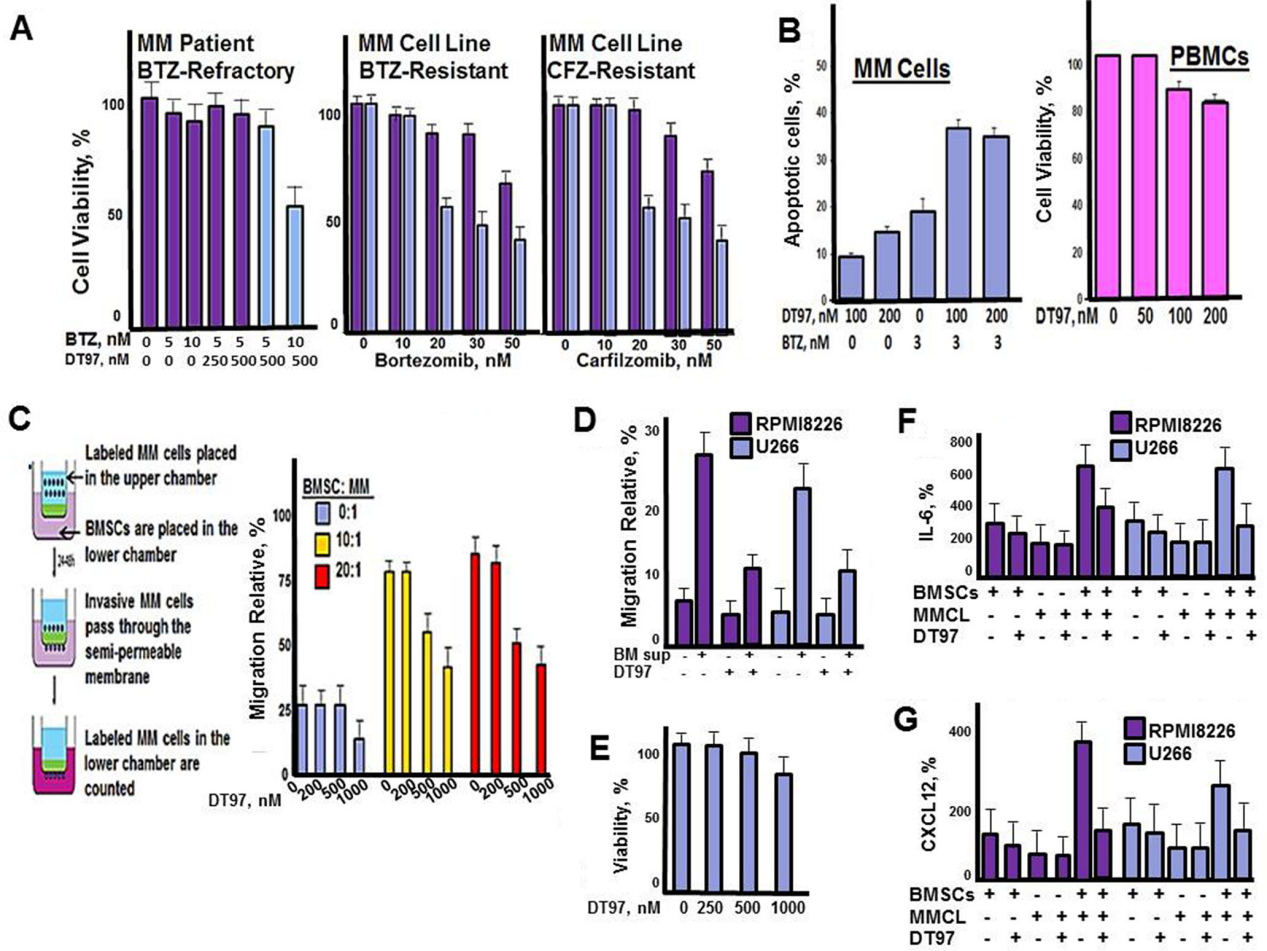
DT97 overcomes bortezomib-resistance and inhibits chemotaxis (**A**) MM patient CD138^+^ PCs or PI-resistant MM cells were incubated with bortezomib and DT97 at the indicated concentrations. Cell viability was determined using the XTT assay. (**B**) MM cells and PBMCs were incubated with bortezomib and DT97 as indicated. Cells were treated as indicated and apoptotic cells counted using the Roche *in situ* cell death detection kit. (**C**) MM patient PCs were CFSE-labeled, placed in the upper chamber and BMSCs in the lower chamber. CFSE-labeled MM cells that migrated to the lower chamber were then counted at 72 h. All assays were performed in triplicate. Values represent the arithmetic mean. Error bars represent the standard deviation. (**D**) Effect of BM sup on the migration of MM cells. Shown is the relative number of MM cells that migrated through transwells with given supplement or treatment. Values represent the arithmetic mean of triplicate measurements. Error bars represent the standard deviation (**E**) Effect of DT97 on BMSCs. After treatment at the given concentration for 72 h, viability was determined using the XTT assay. Values represent the arithmetic mean of triplicate measurements. Error bars represent the standard deviation. (**F**) Effect of DT97 on IL-6 production. After the given treatments, the Quantikine Human IL-6 Immunoassay (R&D Systems, Minneapolis, MN) that employs a solid phase sandwish ELISA was used to measure IL-6 in cell culture supernates, according to the manufacturer's instructions. (**G**) Effect of DT97 on CXCR12 production. After the given treatments, the Human CXCL12/SDF-1 alpha Quantikine ELISA kit (R&D Systems, Minneapolis, MN) was used to measure CXCL12 in cell culture supernates, according to the manufacturer's instructions.

Results indicated that co-treatment with DT97 and either bortezomib or carfilzomib overcame resistance to PIs (Figure 5A). Importantly, results indicated that DT97 affected drug-resistant RPMI8226 cells but did not induce apoptosis in peripheral blood mononuclear cells (PBMCs) (Figure 5B). BMSCs induce the migration and homing (chemotaxis) of MM cells to BM. To investigate the effect of DT97 on chemotaxis, RPMI8226 cells were labeled using the CellTrace reagent CFSE (5(6)-carboxyfluorescein N-hydroxysuccinimidyl, a cell permeant, pro-dye that permits the tracing single cell types in mixed cell type populations. The CellTrace method employs a bright, single-peak green fluorescent stain that enables visualization of tumor cells co-cultured with BMSCs. CFSE covalently binds intracellular proteins and is well-retained in cells for several days, is non-toxic and does not adversely affect growth. Intracellular esterases in live cells then cleave CFSE-acetate to generate the green fluorescent carboxy-fluorescein. MM cells were placed in the upper chamber separated from BMSCs in the lower chamber by a semi-permeable membrane. DT97 treatment reduced the migration of MM cells into the lower chambers with BMSCs (Figure 5C). We then demonstrated that the culture media from BMSCs (BM sup) also promoted the migration of MM cells and that the effect of BM sup on migration was reduced by DT97 treatment (Figure 5D). In addition, treatment with DT97 did not significantly reduce the viability of BMSCs (Figure 5E). Co-culture of MM cells with BMSCs significantly increased the production of the cytokines Il-6 and CXCR12 but the induction was inhibited by DT97 treatment (Figure 5F, 5G). It appears that DT97 treatment prevents MM migration, in part, through the reduction of cytokine production.

### Efficacy of DT97 against other cancer cell types

**Figure 6:**
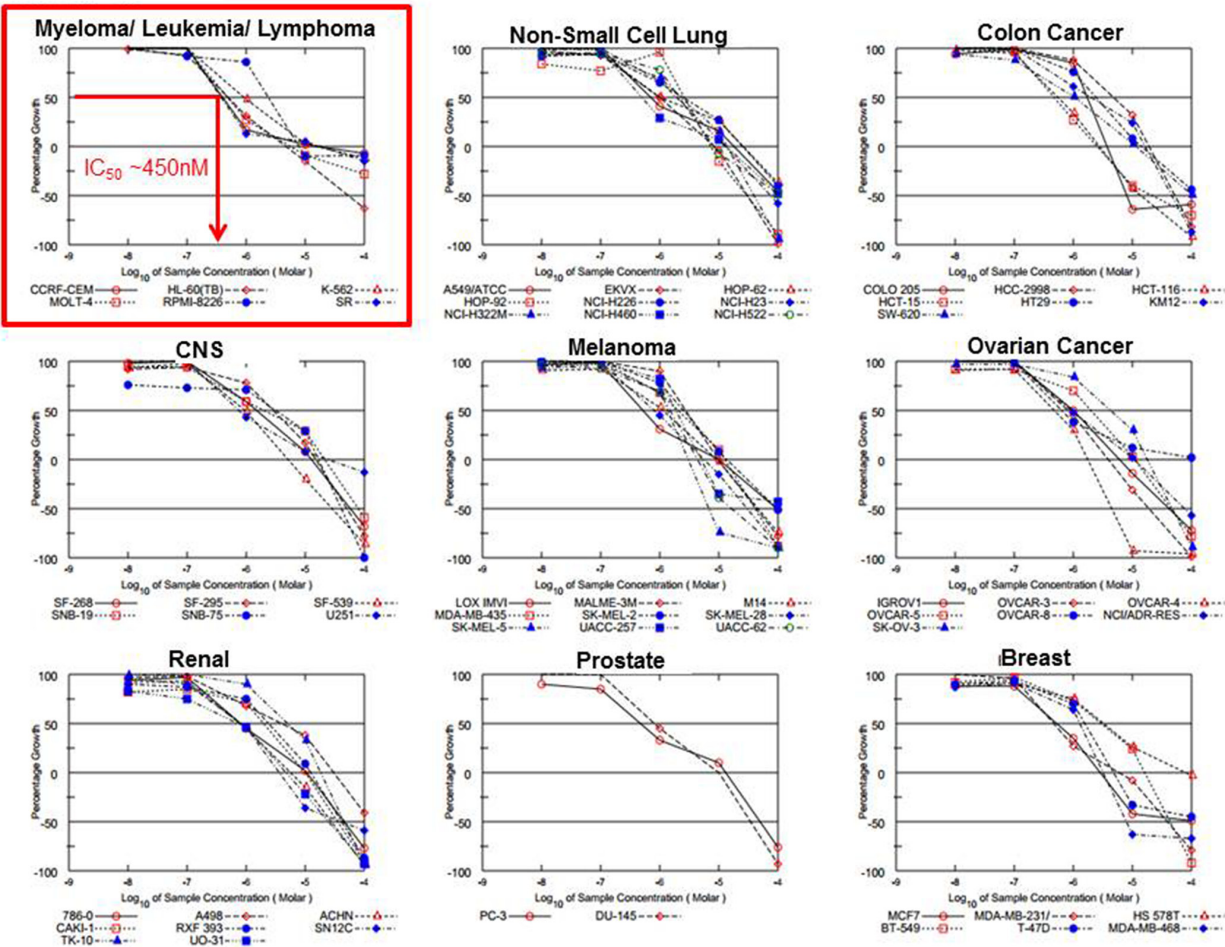
Evaluation of DT97 in the NCI-60 panel The anticancer effect of DT97 in different cancer types was independently determined at the NCI in the Developmental Therapeutics Program. DT97 was tested at the indicated concentrations using the NCI-60 panel of 60 different human solid tumor and blood cancer cell lines to determine the effect on cell growth.

The anticancer effect of DT97 was externally validated using the NCI-60 panel of human cancer cells (Figure 6). The anticancer efficacy of DT97 prioritized to MM, leukemia and lymphoma cancer cell lines (Table 2). The most potent effect was seen in the MM cell line RPMI8226 (IC_50_ ~450 nM).

**Table 2:** Sensitivity of cell lines in the NCI-60 panel to DT97

Percent Growth								
Panal/cell line	−8.0	−7.0	−6.0	−5.0	−4.0	GI50	TGI	LC50
** Leukemia**								
CCRF-CEM	104	106	17	2	−7	4.26E-7	1.60E-5	> 1.00E-4
HL-60 (TB)	99	93	31	−15	−63	4.87E-7	4.69E-6	5.31E-5
K-562	108	111	48	3	−12	9.29E-7	1.65E-5	> 1.00E-4
MOLT-4	111	116	27	9	−28	5.46E-7	5.53E-6	> 1.00E-4
RPMI-8226	101	92	86	−10	−9	2.38E-6	7.90E-6	> 1.00E-4
SR	109	113	13	5	−15	4.26E-7	1.72E-5	> 1.00E-4
** NSCLC**								
A549	106	112	41	16	−46	7.47E-7	1.81E-5	> 1.00E-5
EKVX	97	93	50	−4	−99	1.01E-6	8.55E-6	3.08E-4
HOP-62	93	95	50	26	−37	1.01E-6	2.54E-5	> 1.00E-5
HO-92	84	77	96	−15	−89	2.61E-6	7.38E-6	2.98E-5
NCI-H226	92	94	65	27	−40	2.45E-6	2.54E-5	> 1.00E-5
NCI-H23	98	102	67	7	−58	1.93E-6	1.28E-5	7.59E-5
NCI-H332M	94	94	70	15	−94	2.31E-6	1.37E-5	3.94E-5
NCI-H460	101	98	29	7	−48	4.99E-7	1.37E-5	> 1.00E-5
NCI-H522	95	95	68	−7	−48	2.12E-6	8.17E-6	> 1.00E-5
** Colon cancer**								
COLO205	94	99	85	−64	−59	1.71E-6	3.70E-6	8.03E-6
HCC-2998	105	106	88	32	−82	4.77 E-6	1.91E-5	5.24E-5
HCT-116	98	95	34	−43	−92	5.55E-7	2.80E-6	1.41E-5
HCT-15	100	97	27	−40	−70	4.64E-7	2.50E-6	2.12E-5
HT29	103	108	76	8	−44	2.42E-6	1.44E-5	> 1.00E-4
KM12	104	104	61	24	−87	2.03E-6	1.65E-5	4.66E-5
SW-620	94	88	51	3	−49	1.04E-6	1.16E-5	> 1.00E-4
** CNS Cancer**								
SF-268	98	100	59	8	−68	1.48E-6	1.28E-5	5.77E-5
SF-295	92	94	78	17	−78	2.84E-6	1.50E-5	5.07E-5
SF-539	93	98	49	−20	−86	9.57E-7	5.13E-6	2.85E-5
SNB-19	95	94	59	29	−59	1.98E-6	2.14E-5	7.83E-5
SNB-75	76	73	75	29	−100	3.23E-6	1.69E-5	4.12E-5
U25	103	107	43	8	−13	7.80E-7	2.42E-5	> 1.00E-4

The anti-tumor screening was performed at the National Cancer Institute (NCI) Developmental Therapeutics Program (DTP), Bethesda, Maryland. Compounds were subjected to the NCI's *in vitro* disease-oriented human cells screening panel assay. 60 cell lines of nine tumor subpanels were incubated with five concentrations (0.01–100 mM) of DT97 to create log concentration vs % growth inhibition curves. Three response parameters, median growth inhibition (GI50), total growth inhibition (TGI) and median lethal concentration (LC50), were calculated for each cell line. The GI50 value corresponds to the compound's concentration causing 50% decrease in net cell growth, the TGI value is the compound's concentration resulting in total growth inhibition and the LC50 value is the compound's concentration causing a net 50% loss of initial cells at the end of the incubation period.

### Efficacy of DT97 to induce apoptosis

**Figure 7:**
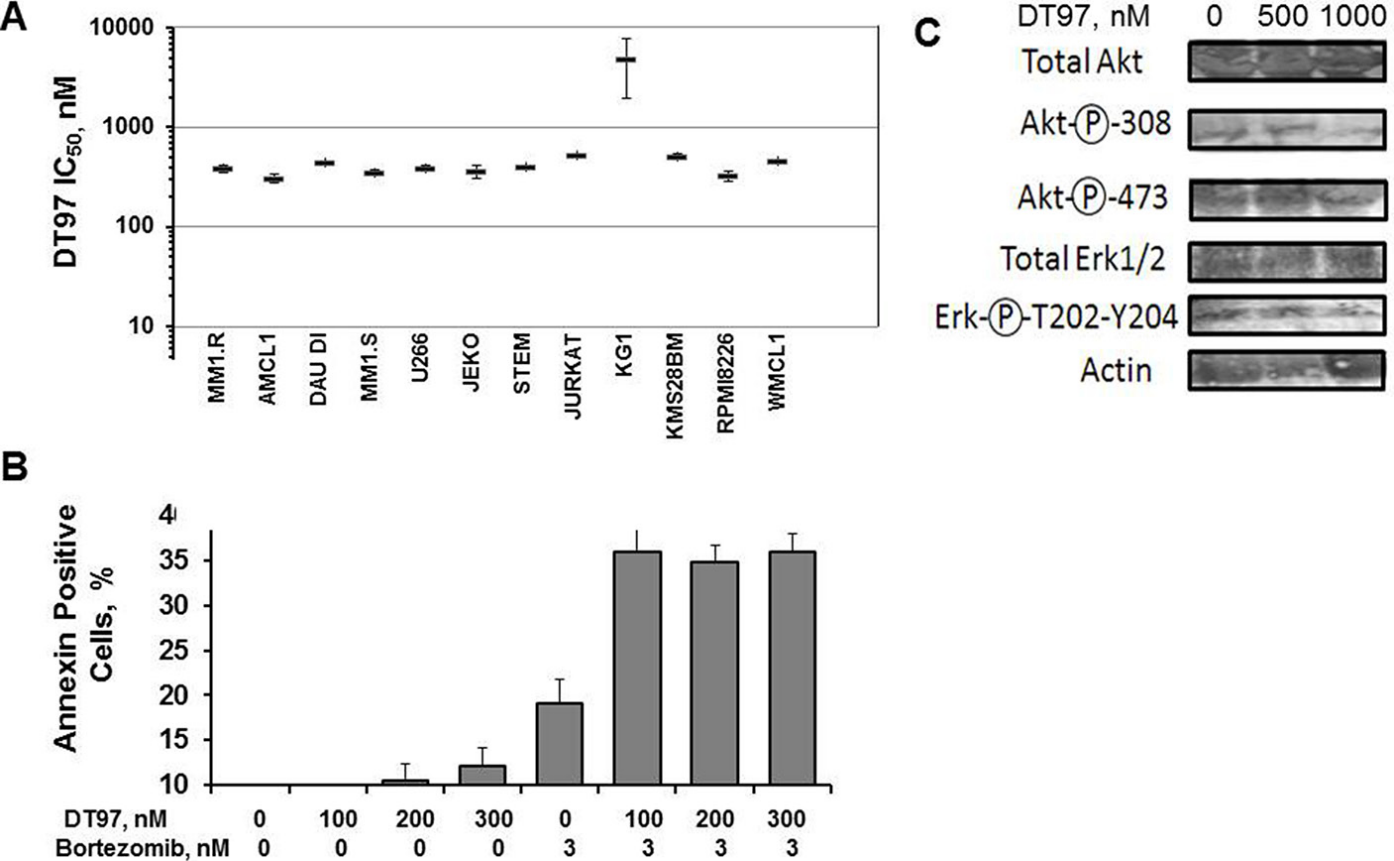
Induction of apoptosis in different hematologic cancer cell lines (**A**) Relative number of annexin-positive cells detected after treatment with DT97 and bortezomib at indicated concentrations. Error bars represent the SD. (**B**) IC_50_ values for DT97 against 12 hematologic cancer cell lines. The effect of DT97 on cell viability was determined using the XTT assay. Values represent the arithmetic mean and error bars represent the SD. (**C**) Effect of DT97 on AKT and ERK phosphorylation. MM cells were treated with DT97 at 500 nm or 1 uM for 48 h and lysates probed using the following antibodies: pan-AKT (Cell Signaling Technology CST4658), phospho-AKT Thr308 (CST4056), phospho-AKT Ser473 (CST3787), total ERK1/2 (pp44/42 MAPK, CST4695) and the phospho-ERK1/2 Thr202/Tyr204 (CST4370).

IC_50_ values for DT97 determined using blood cancer cell lines ranged from 300 to 500 nM (Figure 7A). MM cells were treated with DT97, annexin-positive cells quantitated by flow cytometry and results analyzed using Compusyn software (Figure 7B). The Chou-Talalay theorem quantitatively determines the effect of two drugs added together by determining a combination index (CI). By definition, CI = 1 reflects an additive effect, CI < 1 reflects drug synergism and CI > 1 reflects drug antagonism [24–26]. Co-treatment with DT97 and bortezomib synergistically increased the number of apoptotic cells. CI values for bortezomib at 3 nM and DT97 added at 100, 200 or 300 nM were < 1 indicating that the effect of this combination was synergistic. AKT and ERK1/2 are downstream targets of PI3K. DT97 treatment reduced the phosphorylation of AKT at Ser308/Thr473 and ERK1/2 at Thre202/Tyr-204 (Figure 7C).

### Effect of DT97 on MM patient tumor cells co-cultured with BMSCs

**Figure 8:**
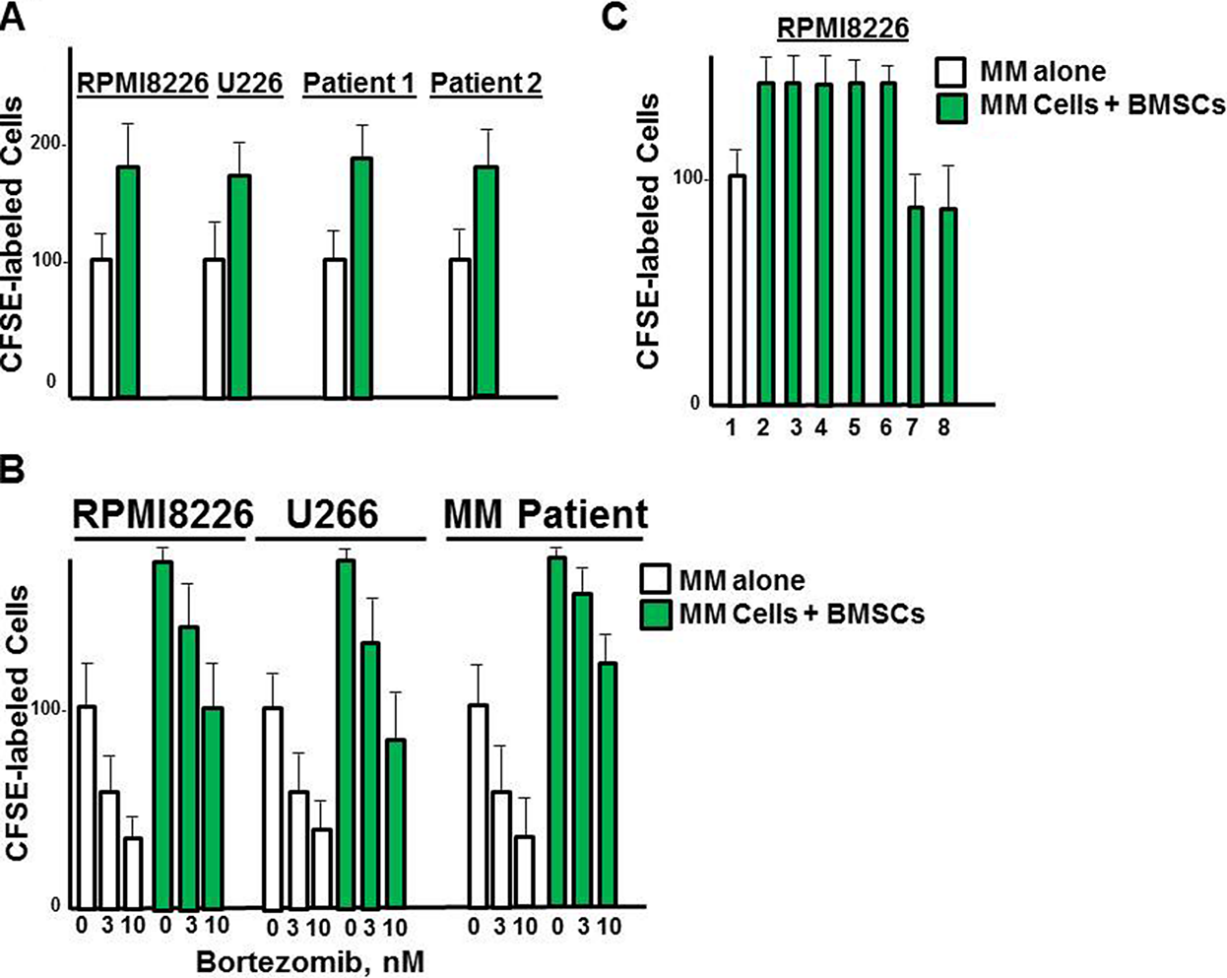
Effect of DT97 on MM cells in the presence of BMSCs (**A**) Effect of BMSCs on the growth of MM cells. RPMI8226 cells were labeled with CellTrace, and cultured alone or with BMSCs. The number green cells was then counted by confocal microscopy. Values represent the arithmetic mean and error bars represent the SD. (**B**) Effect of DT97 on bortezomib sensitivity in the presence of BMSCs. RPMI8226 cells were labeled with CellTrace, and cultured alone or with BMSCs. Cells were then treated with borteomib at indicated concentrations and the number of green cells counted at 72 h. (**C**) Effect of DT97 on MM cells alone or with bortezomib in the presence of DT97. RPMI8226 cells were labeled with CellTrace, treated as indicated and cultured alone or with BMSCs. RPMI8226 cells were cultured alone (lane 1) or with BMSCs (lanes 2–9). Cells were treated as follows: lanes 1, 2- no addition, lane 3- bortezomib 1 nM, lane 4-bortezomib 3 nM, lane 5- bortezomib 5 nM, lane 6- DT97 250 nM, lane 7- bortezomib 3 nM and DT97 250 nM, lane 8- bortezomib 5 nM and DT97 250 nM. Cells were then treated as indicated and the number of green cells counted at 72 h. Assays were performed in triplicate, values represent the arithmetic mean, error bars represent the SD.

A major reason that PI3K inhibitors have been unsuccessful in the clinic may be attributed to a lack of therapeutic efficacy within the tumor microenvironment. CFSE-labeled MM cells were grown in culture with BMSCs and the number of CFSE-positive cells counted at 72 h. The number of RPMI8226 cells was greater in the fraction grown with BMSCs than those grown cells alone (Figure 8A). MM cells grown with BMSCs were also less sensitive to bortezomib than those MM cells cultured alone (Figure 8B). RPMI8226 cells grown with BMSCs were treated with bortezomib, DT97 or both as indicated (Figure 8C). Importantly, CFSE-labeled MM cells grown together with BMSCs were more sensitive to co-treatment with both agents than with either agent alone.

### *In vivo* effect of DT97 as monotherapy and in combination with bortezomib

**Figure 9:**
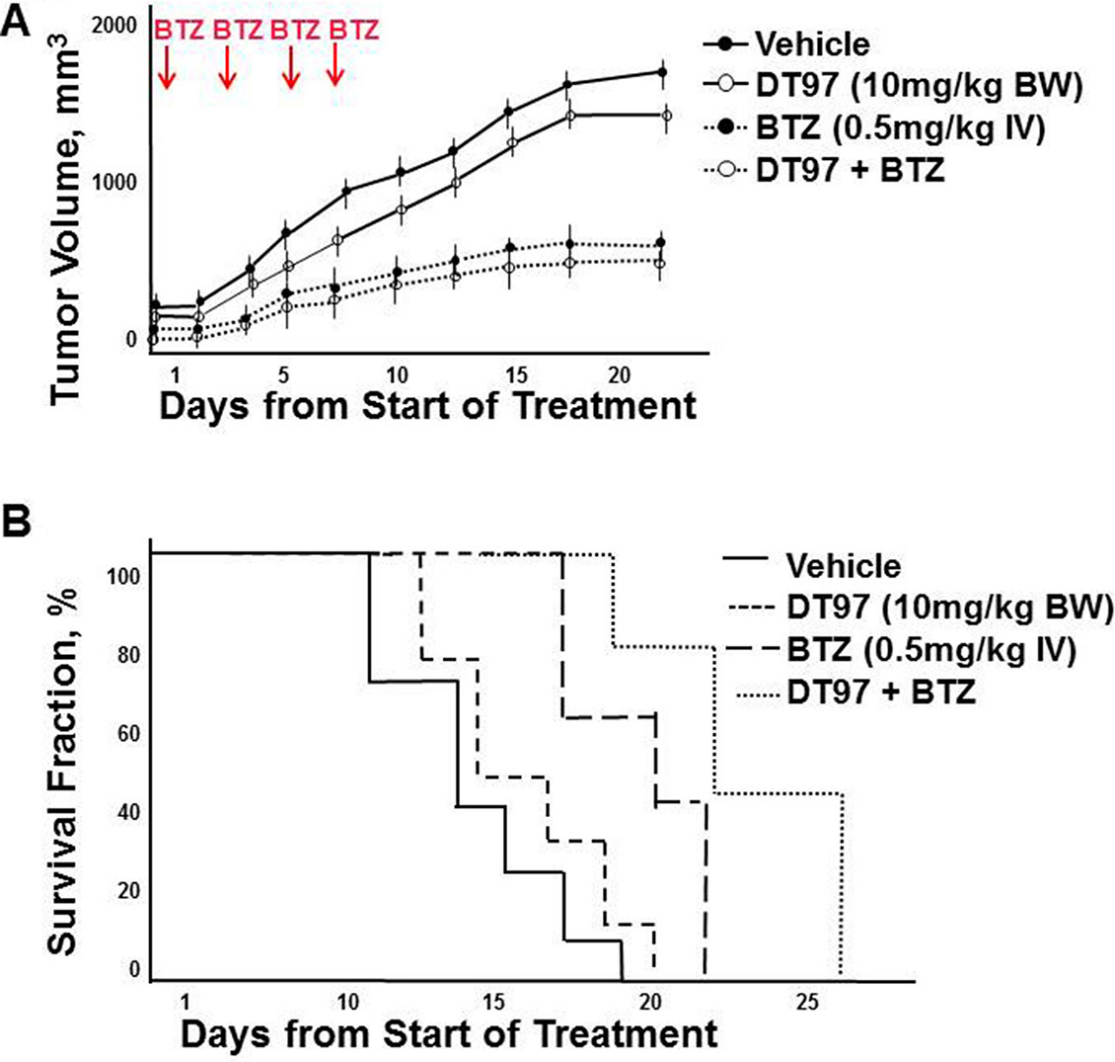
Effect of DT97 and bortezomib on tumor growth and overall survival (**A**) Effect of DT97 on growth of MM tumors *in vivo*. Subcutaneous tumors were generated after injection of myeloma cells. Mice were randomly distributed into four groups (7/group) and received intravenous injection of vehicle (PBS containing 10% DMSO); bortezomib (0.5 mg/kg in PBS, 10% DMSO), DT97 (10 mg/ml diluted in PBS) or both DT97 and Bortezomib. Vehicle or bortezomib was administered on days 1, 4, 8 and 11. Mice were euthanized when tumors reached 2 cm^3^, became ulcerated or elicited neurologic or musculoskeletal complications that limited mobility and feeding. Survival and tumor growth were evaluated from the first day of treatment. Shown is the average of replicate measurements. (**B**) Effect of DT97 on OS. Kaplan-Meier survival curves comparison of NOD. SCID mice after subcutaneous injection of parental myeloma cells treated with vehicle, DT97, bortezomib or both.

Five week old nude mice flanks are injected subcutaneously with MM.1S myeloma cells. Tumors formed within two weeks and mice were then treated intravenously with vehicle, bortezomib, DT97 or both bortezomib and DT97. Treatment with bortezomib alone or bortezomib with DT97 dramatically reduced tumor growth (Figure 9A). Kaplan-Meier survival analysis indicated that treatment with bortezomib and DT97 also improved overall survival (OS) relative those mice treated with vehicle, DT97 or bortezomib alone (Figure 9B). OS for untreated mice was 14 days, DT97 treatment alone was 16 days, bortezomib alone was 20 days and DT97+bortezomib was 23 days (*p* < 0.05).

**Figure 10:**
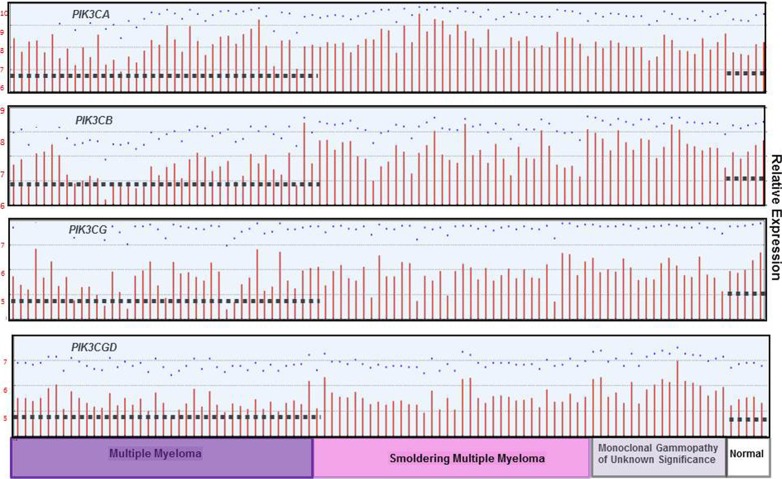
*PIK3C* isoform expression in normal, MGUS, SMM and MM patients The relative expression of each PI3K p110 isoform was determined using dataset GDS4968 in which bone marrow PCs were isolated from patients with monoclonal gammopathy of undetermined significance (MGUS), smoldering multiple myeloma (MM), or MM. The median-centered average of expression is indicated with the dashed line.

**Figure 11:**
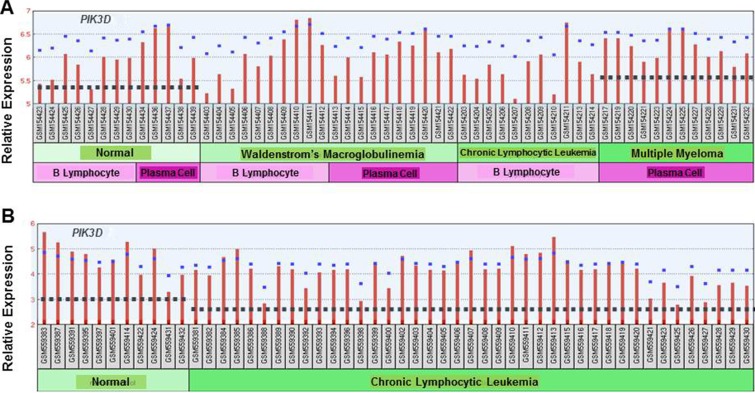
(**A**) Comparison of *PIK3C* isoform expression in normal, WM, CLL and MM patients. The relative expression of the *PIK3CD* isoform was determined using dataset GDS2643 in which B lymphocytes and PCs were isolated from normal patients or those diagnosed with WM, CLL or MM. The median-centered average of expression is indicated with the dashed line. (**B**) Comparison of *PIK3C* isoform expression in B lymphocytes from normal individuals and CLL patients. The relative expression of the *PIK3CD* isoform was determined using dataset GDS4167 in which B lymphocytes were isolated from normal patients or those diagnosed with CLL. The dashed line indicates the edian-centered average of expression.

Publically available datasets were analyzed to compare expression of the different p110 isoforms in MGUS, Smoldering MM (SMM) and MM versus healthy PCs (Figure 10). Dataset GDS4968 compared expression of p110 isoforms from PCs of healthy individuals, MGUS, SMM and MM patients [27]. Importantly, the expression of *PIK3CA, PIK3CB* and *PIK3CG* was actually lower in PCs from MM patients than in healthy PCs. The only PIK3C isoform expressed at greater levels in PCs from MM patients compared to healthy individuals was *PIK3CD*. These findings support the importance of targeting the p110-δ isoform in MM. In addition, dataset GDS2643 was analyzed to compare *PIK3CD* expression in B lymphocytes and PCs from healthy, Waldenstrom's Macroglobulinemia (WM), CLL and MM patients [28] (Figure 11A). Again, *PIK3CD* expression was greater in PCs from MM patients than from healthy individuals. *PIK3CD* expression was also greater in B lymphocytes and PCs isolated from WM patients than from healthy individuals. Moreover, we found that the mean expression of *PIK3CD* was actually lower in B lymphocytes from CLL patients than in B lymphocytes isolated from healthy individuals. Analysis of dataset GDS4167 also supported the finding that *PIK3CD* expression was greater in healthy B cells than those from CLL patients [29] (Figure 11B).

## DISCUSSION

Despite the introduction of PIs and immunomodulatory agents into the clinic, MM remains incurable. While most patients initially respond to PIs, drug resistance emerges and represents the primary factor for treatment failure. Despite the availability of numerous salvage regimens, once drug resistance has emerged, median survival is less than one year. Only ~25% of MM patients respond to bortezomib or carfilzomib when administered as monotherapy, leaving substantial room for improvement. Each PI3K isoform fulfill a unique role in cancer and has prompted development of isoform-selective inhibitors. The lack of efficacy of idelalisib in MM, as well as in acute myeloid leukemia and myeloproliferative neoplasms, is attributed to a highly variable p110-δ active site and a high degree of conformational flexibility that leads to poor inhibitor binding. Structures of p110-δ in complex with a broad panel of isoform- and pan-selective PI3K inhibitors revealed that selectivity toward p110-δ can be achieved by exploiting the sequence diversity and conformational flexibility of the p110-δ active site [11, 30–32]. We used these observations to rationalize the design of a selective p110-δ inhibitor with greatly improved potencies to treat MM. An important downstream effector of PI3K is the Ser/Thr protein kinase AKT, which is activated by phosphorylation of Thr308 and Ser473. DT97 treatment of MM cells reduced AKT phosphorylation at both sites. Previously, AKT phosphorylation was shown to be a major determinant of bortezomib sensitivity [33]. In related studies, bortezomib treatment downregulated phospho-AKT levels in drug sensitive hepatocellular carcinoma cells (HCC) but no alterations of phospho-AKT were observed in drug resistant HCC cells. We speculate that DT97 reduces AKT phosphorylation to overcome drug resistance. Future studies will further investigate the role of DT97 in AKT signaling in MM tumor cells.

Related studies also indicate that treatment of hematologic cancer cells with proteasome inhibitors activates Bruton tyrosine kinase (BTK) [34]. Bortezomib co-treatment with the BTK inhibitor ibrutinib in drug-resistant lymphoma also led to AKT inactivation [35]. The BM microenvironment has also been proposed to promote drug resistance and DT97 treatment effectively reduced MM growth and enhanced the anti-myeloma effect of bortezomib in presence of BMSCs [36]. In summary, we identified a novel small molecule that inhibits p110-δ activity to reduce MM growth and that holds promise for lead compound optimization, pharmacokinetic studies and early phase clinical trials. Combination of DT97 with existing therapies is a rational strategy to improve MM treatment.

## MATERIALS AND METHODS

### Myeloma cell lines and patient tumor cells

MM cell lines (ATCC, Manassas, VA) were cultured in complete RPMI-1640 medium supplemented with fetal calf serum (Hyclone), penicillin-streptomycin, L-glutamine, sodium pyruvate and β-mercaptoethanol (Sigma-Aldrich, St. Louis, MO). AMCL1, AMCL2, leukemic, lymphoma and Waldenström's macroglobulinemia cell lines were cultured as described [37, 38]. Bortezomib and carfilzomib were from Selleck Chemicals (Houston, TX). Human bone marrow stromal cell line HS-5 (ATCC) was cultured in DMEM. Patient bone marrow aspirates were obtained after Institutional Review Board approval or from ConversantBio (Huntsville, AL) and CD138^+^ cells then isolated by microbead selection (Miltenyi Biotec, San Diego, CA). Cells were cultured at 37°C in humidified atmosphere containing 5% CO_2_.

### XTT viability assay

Cells were plated, treated as indicated for 72 h and 2, 3-bis-(2-methoxy-4-nitro-5-sulfophenyl)-2*H*-tetrazolium-5-carboxanilide) added (Sigma-Aldrich) after activation with N-methyl dibenzopyrazine methyl sulfate (Sigma-Aldrich). Absorbance was measured and the effect on myeloma viability determined as previously described [37–39]. Assays were performed in triplicate and the mean and standard deviation determined. Relative cell viability (mean ± SD) was calculated compared to readings from untreated cells.

### Apoptosis assay

Cells were treated as indicated and incubated for 18 h at 37°C. Cells were collected, centrifuged at 200 × g for 5 min, supernatant discarded and the pellet was washed with 1× buffer. Cells were then resuspended in 90 μl of 1 × buffer and 10 μl of FITC-conjugated annexin V (Molecular Probes^®^, Grand Island, NY), incubated in the dark and analyzed using a Coulter^®^ epics^®^ XL-MCL.

### Generation of PI-resistant cell lines

RPMI8226 cells were grown under standard conditions and exposed to successively increased concentrations of the PIs bortezomib, carfilzomib or ixazomib over 6 months to generate PI-resistant cells. As controls, parental cells were exposed to DMSO vehicle under the same algorithm as resistant cells.

### *PIK3C* isoform knockdown

shRNA in pLKO.1-TCR lentiviral cloning vectors were transfected into 293T packaging cells with packaging and envelope vector using lipofectamine 2000 (Thermo-Fisher). Viral supernatants were used to transduce myeloma cells. After 48 h, virus was collected and RPMI8226 cells transduced with vectors expressing scrambled (control) or p110-specific shRNA. Cells were mixed with virus and polybrene (8 μg/mL), centrifuged at 2000 × rpm for 90 min, incubated overnight, media changed and selected in puromycin.

### Quantitation of PI3K activity

To directly assess PI3K activity, PI3K was isolated by immunoprecipitation using an antibody to the p85 adapter subunit and the ability of the co-precipitated catalytic p110 subunit to convert PIP2 to PIP3 in a kinase reaction assessed by measuring the amount of PIP3 generated using a competitive ELISA [40, 41]. 1 × 10^6^ cells were washed thrice with 137 mM NaCl, 20 mM Tris-HCl pH 7.4, 1 mM CaCl_2_, 1 mM MgCl_2_, 0.1 mM Na orthovanadate (Sigma) (buffer A) and lysed in 1 mL of the same supplemented with 1 mM phenylmethylsulphonyl fluoride (PMSF) (Sigma) and 1% nonyl phenoxylpolyethoxylethanol (NP40) (Calbiochem, Darmstadt, Germany) for 20 min on ice. Lysates were centrifuged at 13,000 × rpm for 10 min and supernatants stored at −80°C. Frozen lysates were thawed and PI3K immunoprecipitated with 5 μl anti-PI3K p85 (Cell Signaling Technology, Danvers, MA) for 1 h at 4°C, followed by the addition of 60 μl of a 50% slurry of Protein A agarose beads for 1 h at 4°C. The immunoprecipitate was collected by centrifugation at 13,000 × rpm for 10 sec, washed 3 times in buffer A + 1% NP40, thrice with 0.1 M Tris-HCl, pH 7.4, 5 mM LiCl, 0.1 mM Na orthovanadate and twice with 10 mM Tris-HCl, pH 7.4, 150 mM NaCl, 5 mM ethylenediaminetetraacetic acid (EDTA), 0.1 mM Na orthovanadate. Pellets resuspended in 110 μl of the kinase reaction buffer containing 4-(2-hydroxyethyl)-1-piperazineethanesulfonic acid (HEPES) pH 7.0, 2.5 mM MgCl_2_, 25 μM ATP. Reactions were incubated for 3 h at 37°C with 40 pmol PIP2 (Echelon Biosciences, Salt Lake City, UT). Reactions were stopped with EDTA (5 mM) and centrifuged at 13,000 × rpm at 4°C. Supernatants were transferred to a microtitre plate for a competitive ELISA (Echelon Biosciences) to quantify PIP3 generated. Triplicate reactions were incubated with anti-PIP3 antibody for 1 h at room temperature. Reactions were then transferred to plates coated with PIP3 and incubated for 1 h. After 3 washes with TBS + 0.05% Tween20 (TBST), 100 μl horseradish peroxidase (HRP)-conjugated antibody to the anti-PIP3 was added and incubation continued for 1 h in the dark. Following 3 washes with TBST, 100 μl of tetramethyl benzidine (TMB) substrate was added and reactions were stopped after 20 min with 100 μl 0.5 M H_2_SO_4_. Absorbance was measured (450 nm) and PIP3 quantified using a standard curve generated with experimental samples and plotted on a log scale.

### Mouse xenograft models of myeloma

To evaluate the anti-myeloma effect of DT97 *in vivo*, 5–6 week old female athymic NCr (nu/nu) mice (Charles River, Frederick, MD) were injected subcutaneously with 2 × 10^6^ KMS28BM cells in serum-free medium. After tumors were detected (> 50 mm^3^), mice were randomized to 4 groups (7/group). Mice received intravenous injection of either vehicle (PBS in 10% DMSO); bortezomib (0.5 mg/kg), DT97 (10 mg/kg) or both bortezomib and DT97. Tumor volume (TV) measurements were made using a vernier caliper and calculated using the formula: TV = 0.5 (*a* × *b*^2^) where *a* is the long diameter and *b* the short diameter. Mice were euthanized when tumors reached 2 cm^3^, became ulcerated or elicited neurologic or musculoskeletal complications that limited mobility and feeding.

### Biostatistical analysis

*In vitro* assays were performed in triplicate and data presented are mean ± S.D. of independent experiments performed. Statistical significance of differences (SD) was determined using the Student *t* and ANOVA tests with a minimal level of significance of *P* < 0.05. *In vivo* statistical tests were performed using the 2-tailed Student *t* test. Overall survival (OS) was determined using the Kaplan-Meier method with 95% confidence intervals [42]. Results are presented as the median OS with 95% confidence intervals. Prism Version 5.0 software (GraphPad, La Jolla, CA) was used for statistical analyses.

### Drug synergy

The Compusyn software and program was used to determine the effect of drug combinations and general dose-effects and is based on the median-effect principle and the Chou-Talalay Combination Index-Isobologram Theorem (24–26). The Chou-Talalay method for drug combination is based on the median-effect equation, derived from the mass-action law principle, which provides the common link between single entity and multiple entities, and first order and higher order dynamics. This general equation encompasses the Michaelis-Menten, Hill, Henderson-Hasselbalch, and Scatchard equations. The resulting combination index (CI) theorem of Chou-Talalay offers quantitative definition for additive effect (CI = 1), synergism (CI < 1), and antagonism (CI > 1) in drug combinations.

### Kaplan-meier analysis

*In vivo* statistical tests were performed using the 2-tailed Student *t* test. Median OS was determined using the Kaplan-Meier method with 95% confidence intervals.
